# The Week in the Courts

**Published:** 1911-02-18

**Authors:** 


### The Week in the Courts.

### ILLEGAL OPERATION.

At Hertford Assizes, before Mr. Justice Bucknill, Mr. Sidley Ievers Lightfoot, L.R.C.P., L.R.C.S., of Watford, appeared to answer a charge of performing an illegal operation on a young woman. Defendant, who gave evidence on his own behalf, said that while under examination the girl had a violent eclamptic fit and that in these circumstances his treatment was justifiable. Dr. G. F. Blacker and Mr. Skene Keith gave evidence in support of Mr. Lightfoot's case. The jury returned a verdict of "Guilty" and his lordship sentenced the defendant to five years' penal servitude. Counsel asked for a certificate for an appeal and his lordship said there was abundant evidence to justify an appeal.

### POST-MORTEM EXAMINATION—PARENT'S CONSENT.

In the Court of Session, Edinburgh, before Lord Guthrie and a jury, an action was heard by which one, Boyle, sought to recover damages from Dr. Andrew Love, Ch.B., M.D. Glas., medical superintendent of the Greenock Hospital, for having made a post-mortem examination on the body of Boyle's child, notwithstanding that the father had, so it was alleged, refused his consent. On behalf of Dr. Love it was contended that he was under a statutory obligation to fill up the death certificate accurately, and that the cause of death, in this case, could not be ascertained without an examination, to perform which he did, in fact, obtain Boyle's consent. The jury returned a verdict for the defendant but recommended in future as a general rule that no post-mortem examination should be made without the consent of the nearest relatives.

### ADMINISTRATION OF THE POISON LAW.

One of the most important duties of the Pharmaceutical Society is the administration of the penal provisions of the Pharmacy Acts, and since the facilities for obtaining poisonous preparations have been considerably increased during the past two years, greater viligance is required to ensure that the sale of poisons is conducted in a regular manner. Until April 1909 poisons could only be sold by retail by chemists and druggists, but the Poisons and Pharmacy Act, which came into force at that time, empowered local authorities to license seedsmen, ironmongers and other shopkeepers to traffic in preparations containing nicotine and arsenic for use in horticulture and agriculture, and a large number of licences have been granted under this Act.

Possible new sources of danger have thus been created and additional work has devolved upon the Pharmaceutical Society as a consequence. During last year 1,836 cases of alleged infringements of the Pharmacy Acts were investigated, which is four times as many as in 1908. In 273 cases proceedings were instituted, and in all but a few instances penalties were paid by the persons proceeded against. In the majority of these cases the offences consisted of the sale of poisons by unqualified persons or the keeping open shop for the sale of poisons, but in some cases the offence was non-observance of the formalities which the law requires to be fulfilled in selling poisons. The annual report of the Registrar which was adopted at this month's meeting of the Council of the Pharmaceutical Society shows that the persons from whom it was sought to recover penalties belong to a variety of trading classes, the cases being distributed as follows : 78 drug store proprietors, 3 unqualified managers and assistants in drug stores, 16 chemists, 15 unqualified managers and assistants to chemists, 3 executors of chemists, 3 widows of chemists, 1 unqualified manager to a chemist's executors, 6 limited companies trading as chemists and druggists, 9 unqualified directors, managers and assistants to limited companies, 3 unqualified assistants to medical practitioners, 1 chemical apparatus dealer, 1 medicine-chest fitter, 18 grocers, oilmen and drysalters, 1 horticultural sundriesman, 64 seedsmen, 39 assistants to seedsmen, 7 ironmongers, 2 assistants to ironmongers, 1 manufacturer of a gout specific, 1 sheep dip agent and 1 plumber. The expense incurred by the administration of the penal provisions of the Pharmacy Acts falls on the Pharmaceutical Society, the State contributing nothing towards it.

